# Programming Soft Shape-Morphing Systems by Harnessing Strain Mismatch and Snap-Through Bistability: A Review

**DOI:** 10.3390/ma15072397

**Published:** 2022-03-24

**Authors:** Yi Wu, Gang Guo, Zhuxuan Wei, Jin Qian

**Affiliations:** Key Laboratory of Soft Machines and Smart Devices of Zhejiang Province, Department of Engineering Mechanics, Zhejiang University, Hangzhou 310027, China; 11724012@zju.edu.cn (Y.W.); 11824038@zju.edu.cn (G.G.); 11924009@zju.edu.cn (Z.W.)

**Keywords:** shape-morphing, stimuli-responsive, mismatch, heterogeneity, snap-through bistability, soft actuator, mechanical metamaterial

## Abstract

Multi-modal and controllable shape-morphing constitutes the cornerstone of the functionalization of soft actuators/robots. Involving heterogeneity through material layout is a widely used strategy to generate internal mismatches in active morphing structures. Once triggered by external stimuli, the entire structure undergoes cooperative deformation by minimizing the potential energy. However, the intrinsic limitation of soft materials emerges when it comes to applications such as soft actuators or load-bearing structures that require fast response and large output force. Many researchers have explored the use of the structural principle of snap-through bistability as the morphing mechanisms. Bistable or multi-stable mechanical systems possess more than one local energy minimum and are capable of resting in any of these equilibrium states without external forces. The snap-through motion could overcome energy barriers to switch among these stable or metastable states with dramatically distinct geometries. Attributed to the energy storage and release mechanism, such snap-through transition is quite highly efficient, accompanied by fast response speed, large displacement magnitude, high manipulation strength, and moderate driving force. For example, the shape-morphing timescale of conventional hydrogel systems is usually tens of minutes, while the activation time of hydrogel actuators using the elastic snapping instability strategy can be reduced to below 1 s. By rationally embedding stimuli-responsive inclusions to offer the required trigger energy, various controllable snap-through actuations could be achieved. This review summarizes the current shape-morphing programming strategies based on mismatch strain induced by material heterogeneity, with emphasis on how to leverage snap-through bistability to broaden the applications of the shape-morphing structures in soft robotics and mechanical metamaterials.

## 1. Introduction

Actively morphing flat materials into desired 3D configurations is a promising area of research for functional materials, soft actuators, etc. Multiple applications of stimuli-triggered shape-morphing systems can be found in soft robotics [1,2,3,4], biomedical devices [5,6,7], biomimetic manufacturing [8,9], flexible electronics [10,11,12,13], and mechanical metamaterials [14,15,16,17,18,19]. The spatial arrangement of heterogeneous materials with differential responses to different types of stimuli is a widely used strategy to achieve smart shape transformations [20]. When subjected to external stimuli, the size of the regions occupied by active materials changes greatly while the non-responsive materials retain their original size, and the mismatch strain between these active and passive components drives the entire structure into different 3D configurations, as the consequence of cooperative deformation.

In recent years, soft smart materials [21,22] have attracted great interest due to their advantages of large deformation, compliance, light weight, and the ability to adapt to complex environments. Conventional engineering materials such as metals and ceramics are relatively brittle and easily reach their fracture limits or undergo plastic deformation at small strains. Soft smart materials that respond to various stimuli such as light [23,24], heat [25,26,27], humidity [28,29,30], pH [31,32], and electric field [33] can often reversibly withstand large strains without ultimate failure, making them good material candidates for shape programming. Different stimuli-responsive soft materials such as hydrogels [34,35,36,37], shape memory polymers (SMPs) [38,39,40,41], liquid crystalline elastomers (LCEs) [42,43,44], and dielectric elastomers (DEs) [45,46,47] have been extensively studied in various shape-morphing systems. For high-swelling hydrogels, they often possess good biocompatibility and biodegradability; however, the lack of sufficient mechanical strength and low-speed response limit their use in soft actuators/robots. SMP materials often exhibit good mechanical properties, but their elastic modulus decreases significantly when the temperature becomes high. For SMPs, the pre-programmed temporary shape can be fixed by releasing internal stresses through phase transition, which is a convenient shape-locking mechanism. However, the commonly used SMPs with critical glass transition points only have a one-way shape memory effect, and therefore require reprogramming each time they are used. LCE materials consist of liquid crystalline molecules inside a polymeric network whose alignment can be tuned by external physical fields, resulting in anisotropic dimensional changes and stiffness differences of the materials. DE materials have a fast response speed and large deformation under electric fields, but the main drawback of DEs is their tethered activation, that is, the DE morphing systems are usually equipped with a high-voltage power supply.

The approach of heterogeneous material composition provides a principle for programming the mismatch-induced shape transformations in soft materials [20]. With the in-depth exploration of soft actuators and mechanical metamaterials, these application scenarios require not only the ability to change shape, but also focus on the response time and precise control of the deformation. Considering the intrinsic mechanical properties of hydrogels with low mechanical strength, how to design a fast-actuating soft actuator with large output forces using hydrogel materials remains a challenge. On the one hand, applying proper treatments during material syntheses, such as the use of tough double-network hydrogels [48,49,50], or the addition of magnetic nanocomposites [51,52,53], can compensate for these shortcomings. Furthermore, adopting a snap-through strategy at the structural level can possibly enhance the deformation amplitude and output force [54]. By rationally designing the geometric configuration near the bifurcation point, the slight perturbations caused by external stimuli may enable the structural equilibrium to undergo an abrupt transition, thereby greatly enhancing the response speed. For example, the activation time of a millimeter-scale structure using the elastic snapping instability strategy can be reduced to below 0.6 s [54].

Bistable or multi-stable mechanical systems possess more than one local minimum in the landscape of potential energy, and can rest in multiple stable equilibrium states [55]. A snap-through motion may be induced under suitable stimuli or loading, by which the systems can transit from one stable equilibrium state to another. Such snap-through transition is usually accompanied by significant geometric changes, such as the inversion of shape convexity or post-buckling of slender beams. The vast deformations caused by snap-through do not require external forces to maintain, providing a new shape-locking mechanism distinct from the phase transition within materials. During the snap-through transition in a bistable/multi-stable system, elastic energy is pre-stored in an initial equilibrium state and then rapidly released by overcoming the associated energy barrier. The trigger energy threshold at which the snap-through motion occurs can be quantitatively controlled by harnessing the geometry, which can be employed to achieve amplified shape morphing and/or actuating performances via moderate driving forces.

In this review, we summarize two main strategies of shape-morphing programming. One is based on the mechanism driven by the mismatch strain [56], which can arise from in-plane or across-thickness heterogeneity of material composition. In-plane mismatch strain induces internal stresses and causes out-of-plane buckling, while stress gradients through the thickness direction create out-of-plane bending. Advances in additive manufacturing techniques give access to construct morphing structures through the spatial arrangement of multiple materials. The other programming strategy is the snap-through mechanism, which can further broaden the applications of shape-morphing structures in soft robotics and mechanical metamaterials. Rational combinations of active and passive materials in a bistable structure provide a trigger switch to overcome the bistable threshold tuned by geometry, enabling precise and reliable control of the snap-through transition. The representative shape transformations based on these two strategies, as well as their characteristics and applications, are summarized in Table 1. The design principles of mismatch strain and snap-through bistability can be integrated to form a more practical and sophisticated design for future functional shape-morphing systems.

## 2. Heterogeneous Material Composition

To build up internal stresses within materials, the most convenient way is to introduce stimuli-responsive materials in an initially flat configuration through spatial material arrangements. These active elements can expand or shrink in size in response to external stimuli. In contrast, the passive zone beyond these active elements tends to retain its original shape. These two opposite trends result in distributed strain mismatch within the material structure, driving the shape-morphing process through cooperative deformation. By differently programmed layouts of the active and passive materials in the plane, desired internal stresses can be created upon activation, resulting in out-of-plane buckling when the strain mismatch exceeds a critical value. Similarly, the inhomogeneity of materials along the thickness direction may induce strain mismatch layer by layer, resulting in out-of-plane bending deformation.

### 2.1. In-Plane Inhomogeneity

**Figure 1:**
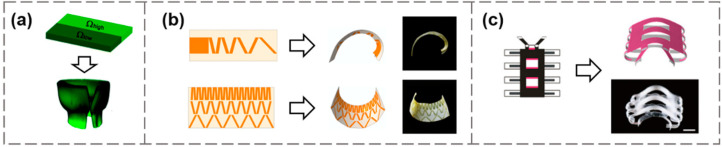
Diverse shape-morphing caused by discretely patterned in-plane inhomogeneities in an initially flat configuration. (**a**) A bi-gel strip with differential swelling ratios rolls into a nearly cylindrical shape after swelling. Reproduced from Ref. [57]; (**b**) Stretching-induced surfaces with different curvatures. Reproduced from Ref. [72]; (**c**) The crystallization pattern and deformed geometry of a worm-like shape. Reproduced from Ref. [58].

Photolithography is one of the most common fabrication methods to tessellate hydrogels with different swelling ratios in a single-layer structure [65,66,67,68]. Local photo-polymerization guided by photomasks can generate heterogeneous hydrogel structures with different degrees of crosslinking. The swelling ratio and Young’s modulus of the gels depend on the amount of exposure. For example, Kim et al. prepared a bi-hydrogel strip with two parallelly arranged regions by photo-polymerization, leading to high-swelling and low-swelling regions [57]. During swelling in an aqueous environment, the length of the high-swelling portion tended to expand, in competition with the smaller dimensional changes of the low-swelling portion. By minimizing the potential energy, the entire structure finally adopted a rolled-up shape, consisting of two cylindrical regions connected by a transition neck (Figure 1a). Ma et al. fabricated hydrogel sheets with periodic swelling variations using a multi-step photolithography method [69] and achieved a 3D wavy reconfiguration when the temperature was increased from 20 °C to 45 °C.

Three-dimensional printing is a powerful manufacturing method used to obtain spatially patterned smart structures. For example, Jin et al. improved the self-supporting printability of a NIPAAm-based thermo-responsive hydrogel system by adding laponite nanoclay [70]. Graphene oxide (GO) was also added as a nanoscale heater in response to near-infrared (NIR) radiation. A series of printed 2D circular-patterned hydrogels transformed into 3D saddle and fan shapes when immersed in a hot bath or irradiated by NIR. Huang et al. proposed ultrafast digital light processing (DLP) printing to precisely control differential hydrogel properties using digitally defined exposure times [71]. Longer exposures resulted in tighter crosslinks that correspond to a lower swelling ratio and a larger modulus. The non-exposed areas were not crosslinked, eventually forming void regions. A representative 3D-theater configuration was designed through this spatially selective digital printing.

SMPs are polymeric networks that have been frequently explored for shape-morphing structures. A dynamic covalent polymer network with flexible programmability was established to realize the spatiotemporal manipulation of the structural topology [72]. The network polymer can be programmed into numerous parts with differently defined thermomechanical properties. Anisotropic dimensional changes were introduced through pre-stretching, and residual stresses were released in specific regions by photocuring. Several 2D topological patterns were programmed to obtain 3D surfaces with different curvatures (Figure 1b). Using a similar approach, Kohlmeyer et al. reported a strategy for shape-reprogramming in a single polymer material that allowed erasing and re-encoding of shape information through reversible localized chemical reaction [73]. Peng et al. reported a digital printing method based on a light-coded process to achieve bioinspired shape transformation [58]. Pre-stretched 2D sheets with spatial heterogeneity in crystallinity were demonstrated to morph with time into designable 3D permanent shapes, enabling 4D transformation [58]. The crystallization pattern and deformed geometry of a worm-like shape are illustrated in Figure 1c.

**Figure 2:**
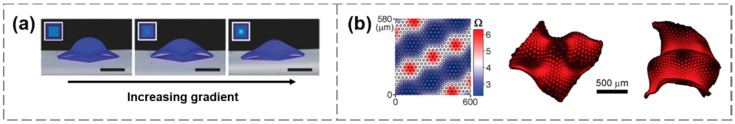
Programming the shape-morphing using in-plane material gradients. (**a**) The cap geometry controlled by increasing gradient distribution. Reproduced from Ref. [71]; (**b**) Multi-stable configurations of a continuous swelling pattern through secondary crosslinking. Reproduced from Ref. [74].

Different from the strategy of discrete patterning, the smooth gradient pattern can also be employed to induce shape-morphing. Figure 2a illustrates the digital printing process for forming a swelling gradient within a planar sheet [71]. The sharpness of the cap tip was adjusted by changing the area exposure time to increase the swelling gradient within the material. Another work used two photomasks for secondary curing cross-linking to embed highly cross-linked dots in a lightly cross-linked matrix [74], resulting in a nearly continuous internal stress gradient (Figure 2b), where Ω was the areal expansion ratio. For this periodic in-plane swelling pattern, the probability of each periodic structural element buckling upward or downward was exactly the same, and the final deformed shape was influenced by prescribed perturbations or geometric defects. The results showed two stable deformed configurations corresponding to the same swelling pattern that appeared in the experiment [74]. To locally control the buckling direction, Wang et al. presented a site-specific pre-swelling method that used masks with holes to generate a transient through-thickness gradient to guide the buckling during the subsequent unmasked swelling process [75].

### 2.2. Through-Thickness Inhomogeneity

Compared with the uncertainty in the buckling direction introduced by the in-plane stress distribution, strain mismatch in the thickness direction provides better control for shape-morphing. When the ratio of in-plane dimension to thickness is large, the two-dimensional plane degenerates into a slender beam. Through-thickness stress gradient tends to generate out-of-plane bending moment, and drives the structure to bend towards a certain direction in response to external stimuli.

As a classical bending problem, Timoshenko established the first theoretical foundation to calculate the curvature of bi-material strips under the mismatch strain induced by uniform temperature variation [76]. Considering a bilayer structure with a unit out-of-plane thickness (i.e., $t_{10}=t_{20}=1$). The initial heights of the expansion and non-expansion layers are $w_{10}$ and $w_{20}$, respectively. The expansion layer and non-expansion layer have their initial moduli of $E_{10}$ and $E_{20}$. The explicit expression for the bending curvature ρ of the bi-strip induced by mismatch strain $\varepsilon_{m}$ can be derived from Timoshenko theory, namely,

(1) $$\rho =\frac{1}{6\varepsilon_{m}}\left[3\left(w_{10}+w_{20}\right)+\frac{E_{10}w_{10}^{3}+E_{20}w_{20}^{3}}{w_{10}+w_{20}}\left(\frac{1}{E_{10}w_{10}}+\frac{1}{E_{20}w_{20}}\right)\right],$$

However, Timoshenko’s analysis on bi-strip bending was based on the assumption of small deformation, and cannot be directly applied to predict the large-magnitude morphing behavior of swelling/non-swelling bi-strips made of soft materials, such as those in hydrogels. Wu et al. proposed a modified model to quantitatively predict the swelling-induced bending of bi-hydrogel strips, in which the differential swelling capabilities and mismatch strain in the composite structure drive the strips into a bending configuration under exposure to solvents [77]. The effect of material softening due to the mixing effects of polymers and solvents was also considered in the analysis [77].

**Figure 3:**
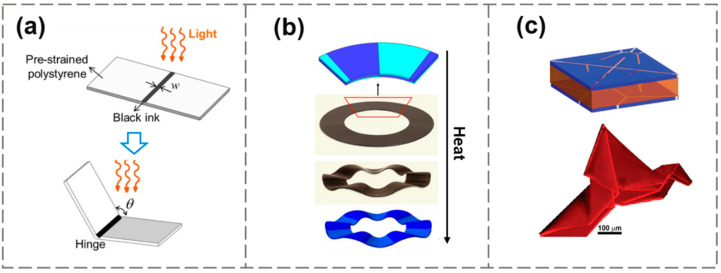
(**a**) Bilayer bending hinge. Reproduced from Ref. [78]; (**b**) Bilayer wrinkling ring. Reproduced from Ref. [79]; (**c**) Tri-layer origami bird. Reproduced from Ref. [59].

On the basis of these theoretical models, Zhang et al. designed a controllable light-responsive self-folding hinge by embedding the bilayer SMP into the structure (Figure 3a) [78]. Only the responsive joint was designed to be deformable, and the other regions performed rigid body motion. With the light-triggered hinge, origami-inspired self-folding structures with different Gaussian curvatures were realized, starting with initially planar polymer sheets [78]. Ding et al. used inkjet printing to obtain active composite materials to obtain high-resolution 3D reprogrammable structures [79]. As illustrated in Figure 3b, a hollow ring with an alternating bilayer layout was shown to transform into a wavy shape after being heated. Na et al. demonstrated a reversibly self-folding origami based on micro-patterned tri-layer films [59]. A thermo-responsive hydrogel layer was sandwiched by thin rigid polymer layers with creases, achieved by photolithography. A microscopic origami bird was made by programming the tri-layer of a photo-crosslinkable copolymer (Figure 3c).

**Figure 4:**
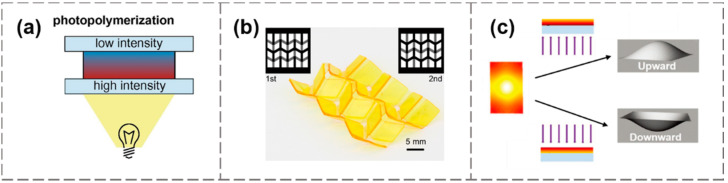
(**a**) Generating material gradients along the thickness direction via light-attenuated photo-polymerization. Reproduced from Ref. [20]. (**b**) Miura folding deformation obtained by double-side illumination. Reproduced from Ref. [80]. (**c**) Controlling the buckling direction by unilateral illumination. Reproduced from Ref. [81].

In addition to the approach of combining multiple smart materials into a multilayer structure, a customized gradient of material properties in a mono-layer structure can also be used to generate a stress distribution along the thickness direction. Such in-plane patterning can avoid the risk of debonding and delamination that may occur at the heterogeneous interface [20]. A commonly adopted treatment for generating in-plane patterning is to use the penetration attenuation characteristics of light to illuminate from one side to create a light intensity gradient across the thickness, thereby affecting the crosslinking density of the polymer, as shown in Figure 4a. Based on this method, Zhao et al. developed a double-side photo-polymerization process by irradiating through differently patterned photomasks from the upper and lower sides [80], through which Miura folding deformation was realized (Figure 4b). Zhou et al. extended the approach by changing the direction of illumination within hydrogel layers [81], resulting in a controllable planar patterning that generated buckling behavior (Figure 4c). Different from Wang’s pre-swelling strategy to control the buckling direction [75], this method introduced the stress gradient into the material during the manufacturing process, and the direction of morphing was prescribed. Besides photo-polymerization, electrophoresis and ionoprinting [82] are alternative ways to generate stress gradients along the thickness direction of the material.

**Figure 5:**
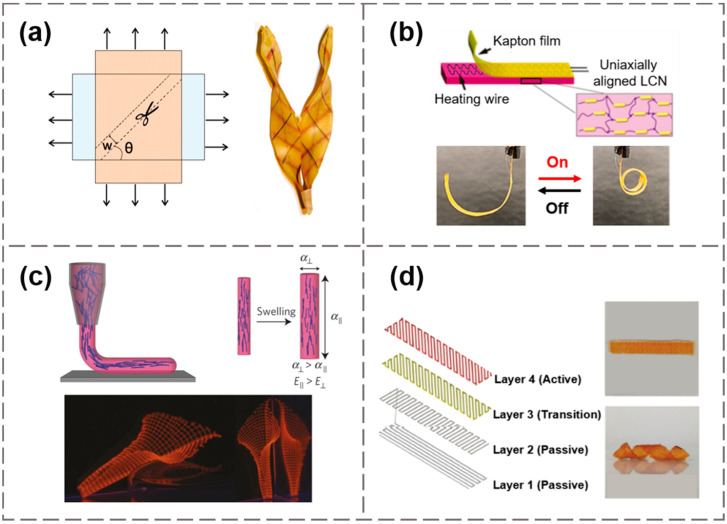
(**a**) A pre-stretching induced pod-like chiral shape. Reproduced from Ref. [84]; (**b**) An LCE rolled-up actuator. Reproduced from Ref. [60]; (**c**) Biomimetic structures printed by anisotropic hydrogel filaments. Reproduced from Ref. [9]; (**d**) Helical shape generated by different alignments of active gel fibers in a hydrogel composite. Reproduced from Ref. [61].

Generating anisotropic material responses is another effective way to produce complex shape transformations. By inducing stresses in the desired direction, such as the pre-stretching of elastomers, some deformation characteristics can be decoupled from the initial 2D geometry [83]. In a study by Armon et al., two planar latex sheets were stretched uniaxially along two perpendicular directions and then glued together, forming a residually stressed composite sheet [84]. As a strip was cut off this composite sheet along a particular direction that formed an angle with one of the stretching directions, an interesting pod-like chiral shape transformation was achieved [84], as illustrated in Figure 5a. Compared with ordinary elastomers, SMPs can lock the pre-stretched state as a temporary shape, and recover to the original shape upon proper activation. If a piece of SMP film is biaxially pre-stretched, its recovery process can be approximated as isotropic shrinkage. By cyclically stretching LCEs, the orientation of liquid crystal molecules can be trained to be aligned along the stretch direction. As shown in Figure 5b, Xiao et al. designed an electrically driven actuator by embedding resistance wires between polyimide films and LCEs [60]. The orientation of the liquid crystal network can be changed by pre-stretching, thereby realizing multi-modal deformation modes of bending and twisting.

To realize localized programming of anisotropy through extrusion printing, anisotropic fillers were added to the printing ink, allowing precise spatial control of material properties such as elastic modulus (*E*) and coefficient of expansion (*α*) [9]. The shear-induced alignment of anisotropic fillers at the nozzle will define the localized anisotropy via pathway control. Gladman et al. printed several biomimetic shapes with hydrogel composite ink composed of stiff cellulose fibrils embedded in a soft acrylamide matrix [9]. The printing path dictated the local orientation of the cellulose fibrils, i.e., the localized swelling anisotropy. They designed shape morphing with controllable Gaussian curvatures using a bilayer lattice structure, as shown in Figure 5c, and obtained a swelling-induced lily-like architecture. Following the approach, Boley et al. adopted a printable ink system, including polydimethylsiloxane (PDMS) elastomeric matrices with tunable glass fibers [85], and a 3D human face was realized by anisotropic expansion/shrinkage of multiplexed bilayer ribs. In addition, by changing the oblique angle of printing path, anisotropic swelling can also be achieved with isotropic hydrogel inks by forming structure-level features [61]. As shown in Figure 5d, a helical shape change was obtained by multi-layered hydrogel composites in which each layer was printed with different responsiveness and printing paths, resulting in programmable 4D morphing.

## 3. Structural Instability

Structural instability refers to the phenomenon that a given structure loses its stable state when the applied load or external stimuli increase beyond a threshold value. Conventionally, such instability is regarded as a type of failure, which should be avoided in the reliability design of engineering structures. In the past two decades, researchers have realized the merits of involving instabilities in soft shape-morphing systems, and began to apply structural instabilities in the design of soft actuators and mechanical metamaterials. Instability-induced morphogenesis, including buckling, twisting, wrinkling, and creasing, enriches the possibilities of bionic shape transformations. In particular, the snap-through motion between bistable states in a nonlinear system is favored due to the rapid transition between energy storage and release. The structure-based instability strategy is scale-free and material-independent, and is complementary to the design of functional morphing systems.

### 3.1. Energy and Stability of Shape-Morphing Structures

**Figure 6:**
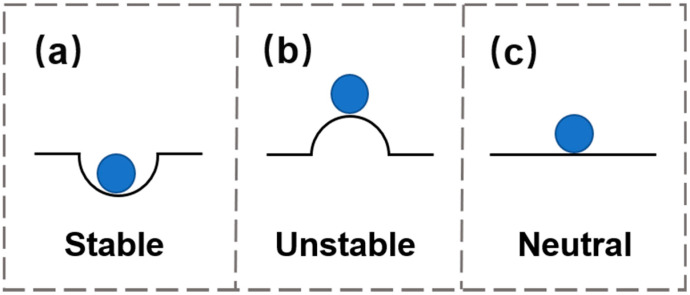
Schematic diagram of structural stability in an energy landscape. (**a**) stable; (**b**) unstable; (**c**) neutral.

When subjected to an external stimulus, the spatially heterogeneous membrane structure transforms into the desired 3D shape by varying its energy in coupling with the external environment. Considering that the total potential energy consists of the internal strain energy and the energy from the external stimulus, namely,

(2) $$U_{t}=U_{s}+U_{b}+U_{e},$$

where $U_{s}$ is the stretching strain energy, $U_{b}$ is the bending strain energy, and $U_{e}$ is the energy absorbed from external loads. According to the principle of minimum potential energy, the equilibrium state is defined by the condition:

(3) δU=0,

In general, several solutions can be obtained by solving the condition that the variation of the total potential energy is equal to zero, and some of them may not be stable. In analogy to the system that when a ball rests on top of a sharp hill (Figure 6b), it is in equilibrium, but any small perturbation will break the balance and result in an irreversible change of the ball’s state. In contrast, if the ball is placed at the bottom of a valley, the equilibrium state is stable (Figure 6a) because the ball tends to return to the original position in the presence of external perturbations. The third circumstance is called neutral equilibrium (Figure 6c), described by the scenario that the ball is placed on a flat surface and the total potential energy is invariable. To further understand the stability of the structure, the convexity or concavity of the total potential energy profile should be investigated. The system is stable if the second derivative of the total potential energy is $\delta^{2}U>0$, unstable if $\delta^{2}U<0$, and neutral if $\delta^{2}U=0$.

(4) $$\delta^{2}U\{\begin{array}{c} >0,\;\;\;\;\;\mathrm{stable}\\ =0,\;\;\;\;\mathrm{neutral}\\ <0,\;\;\;\mathrm{unstable} \end{array}$$

For the deformation of a 2D shell structure made of the material with Young’s modulus E and Poisson’s ratio v, the strain energy is composed of two parts: bending and stretching. The stretching part can be scaled as $U_{s}\sim K\int^{\;}\varepsilon^{2}d\omega$, where $K=Eh/\left(1-v^{2}\right)$ is the stretching rigidity, ε is the stretching strain, and dω refers to the areal element. It can be seen that $U_{s}$ is linear to the sheet thickness h and corresponds to the in-plane deformations. The bending energy can be scaled as $U_{b}\sim D\int^{\;}\kappa^{2}d\omega$, where $D=Eh^{3}/\left[12\left(1-v^{2}\right)\right]$ is the bending rigidity and κ is the bending strain of the middle surface of the sheet. $U_{b}$ depends on $h^{3}$ and accounts for the change in out-of-plane curvature. The strain energy of the thin shell can be represented as

(5) $$U\sim \overset{K\int \;\varepsilon^{2}d\omega }{\underset{U_{s}}{⏟}}+\overset{D\int \;\kappa^{2}d\omega }{\underset{U_{b}}{⏟}},$$

notice that

(6) $$U_{b}/U_{s}\sim h^{2}{\left(\kappa /\varepsilon \right)}^{2}$$

for the shell structure, the thickness *h* is much smaller than the other dimensions. Therefore, it is easier for the shell to bend rather than stretch in terms of energy cost, and these thin 2D shell structures are prone to instability.

The competition between bending and stretching leads to instability of the structure, which has been extensively investigated for the shape-morphing of soft matter. Instability-induced morphogenesis widely exists in nature, such as in the rapid closure snap mechanism of Venus flytrap leaves [86], the different short-wavelength edge wrinkling of Lotus leaves [87], and the wrinkling of pumpkin surfaces [88]. This plant morphogenesis is induced by instability, including buckling, wrinkling, creasing, and snapping, providing inspiration for biomimetic morphing structures. However, instability always comes with strong nonlinearity at the structural level, even if the material remains in the near-linear region, and how to precisely control the shape-morphing caused by instabilities in nonlinear structures remains a challenge

### 3.2. Bifurcation and Snap-Through Instability

**Figure 7:**
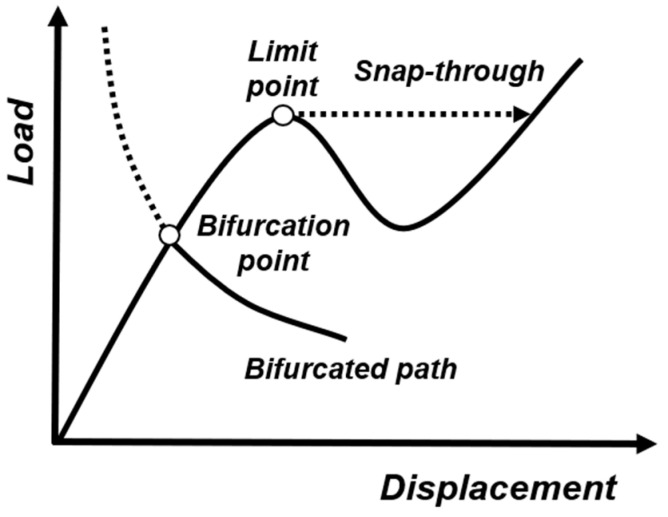
Bifurcation and snap-through point in a representative force-displacement curve.

There are typically two critical points for the instability occurring in elastic structures: the bifurcation point (or buckling point), and the limit point (or snap-through point), as depicted in Figure 7. The bifurcated branch intersects the fundamental path (branching) at a bifurcation point, meaning that two or more equilibrium states exist [89]. On the other hand, a snap-through point occurs when the equilibrium position becomes unstable, and the structure state jumps to the closest stable point on the same equilibrium path. The difference between these two types of instability scenarios is the following: a snap-through point follows only one equilibrium path without changing the deformation form, while a bifurcation point involves a second equilibrium path, requiring the exchange of stability between two equilibrium paths. Holmes [90] created a vivid analogy stating that the snap-through motion is similar to a puddle one must jump over to continue when walking on a path in the woods, while the bifurcation is a fork in the road where a new path emerges.

Bifurcation and snap-through instability occur when the applied load or external stimuli increase beyond a threshold value. During the bifurcation/snap-through process, large deformations or dramatic changes in geometry arise within a short period of time (tens of milliseconds) due to the sudden release of stored strain energy, resulting in relatively large output forces at relatively low input energies. The initial geometry of a bistable structure affects the energy barrier between the two states, providing a quantitative method for controlling snap-through motions, which is promising in shape-morphing of actuators. Also, smart materials can be embedded into bistable structures as a switch of the actuation, offering trigger energy under remote control.

### 3.3. Snap-Through Bistability in Soft Actuators

**Figure 8:**
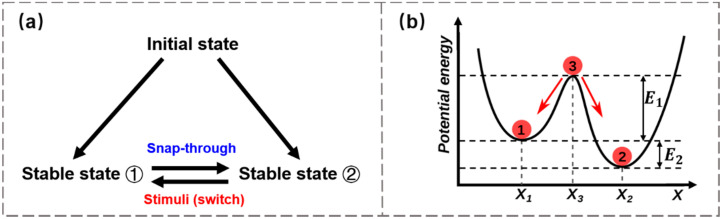
Snap-through bistability. (**a**) Embedding smart materials into bistable structures to trigger the snap-through transition and the switch between the two stable states; (**b**) Representative scheme of potential energy as a function of displacement for bistable structures (state 1 or 2: stable; state 3: unstable).

The soft actuator is one of the main application scenarios for functional soft morphing systems to perform specific tasks. Due to the intrinsic characteristics of soft materials, mismatch-induced shape-morphing typically exhibits slow locomotion speed and low strength, limiting their use in practical applications. Actuators taking advantage of snap-through instability may achieve higher performances, with larger output forces and faster responses, than conventional actuators through rapid energy storage and release. For a typical bistable system, the two stable equilibrium states usually correspond to different configurations in geometry, suggesting that the snap-through transition can cause remarkable shape change. Such bistable systems require relatively low energy inputs to trigger the actuation. There is also no need to provide continuous inputs to maintain the final shape once the actuation is complete. These inherent features of bistable structures render them a promising strategy for their use in soft actuators. With a rational design, a variety of actuators and robotics with snap-through instability have been explored to achieve large deformations, and stimuli-responsive materials have been integrated with the bistable structures to provide moderate actuation forces [33,54,62,63,64]. Controllable snap-through actuation occurs when a suitable stimulus offers the trigger force to sufficiently overcome the energy barrier between different stable states, as shown in Figure 8a. In a representative potential energy-displacement curve like the one shown in Figure 8b, either state 1 or 2 is at a local energy minimum (stable). Once a trigger stimulus is imposed, the entire system accumulates energy until the critical energy threshold $E_{1}$ (for state 1 to 2) is reached, and then rapidly releases energy $E_{1}+E_{2}$, jumping to the stable state 2.

**Figure 9:**
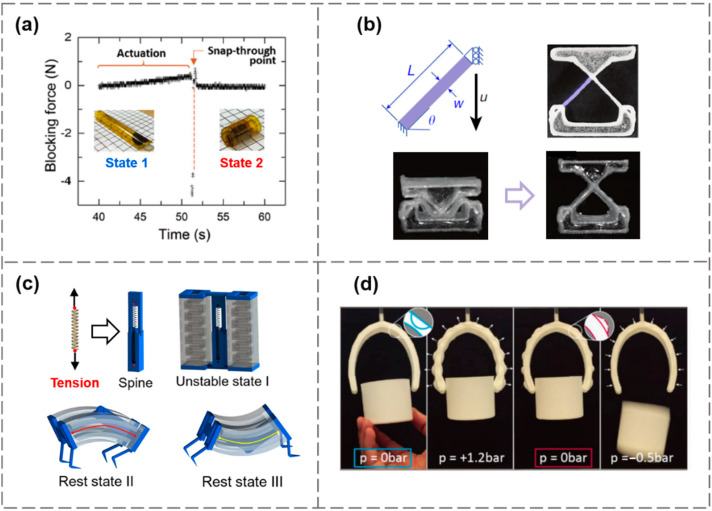
Soft actuators inspired by snap-through bistability. (**a**) A DEA mimicking insect capture. Reproduced from Ref. [33]; (**b**) The rapid snap-through actuation of a hydrogel composite with the initial geometry near the bifurcation point. Reproduced from Ref. [54]; (**c**) A spine-inspired bistable soft pneumatic actuator. Reproduced from Ref. [62]; (**d**) A pneumatic gripper with a dome-patterned outer skin. The reversible inversion of domes causes a grabbing force on an object, even without the external pressurization. Reproduced from Ref. [63].

Elastic structures, such as 1D rods, beams, and 2D shells, membranes, all exhibit snap-through bistability, which can be used for the structural basis in designing soft actuators. For example, Shao et al. reported a bioinspired tri-layer dielectric elastomer actuator (DEA) using a bistable structure with snap-through instability, mimicking the process of insect capture by a frog [33]. In their work, the energy was stored in the structure by biaxial stretching the top and bottom DE layers, which were bounded to the intermediate support layer. The initial equilibrium state of this sandwich structure tended to bend along the short side. Under the action of an external electric field, the DE actuator suddenly transformed to a rolled-up shape. The activating area coated with a compliant electrode only occupied a small fraction of the overall structure, but was sufficient to drive remarkable shape change. Furthermore, there was no need for the voltage to be continuously applied to maintain the deformation of the sandwich structure. The force generated by the tape-spring actuator was measured, as shown in Figure 9a, where the blocking force refers to an external equivalent force applied to counteract the electric field-induced deflection. The change of blocking force over time reveals that the snap-through motion took about 0.2 s, and the full actuation was completed in 1.7 s. Jiang et al. fabricated laterally-constrained beam composites with their initial geometries near the bifurcation points for a transition between bistability and monostability [54]. As demonstrated in Figure 9b, the slenderness ratio w/L of the tilted beam is one of the key parameters to determine whether or not the structure possesses the bistability feature. Direct ink writing (DIW) of glass fiber composites was adopted to precisely control the value of w/L. Once undergoing anisotropic swelling, a bifurcation point was crossed and the triggered snap-through motion was accompanied by rapid and large amplitude self-actuation. By properly tuning the size of the energy barrier, the actuation time of millimeter-scale structures can be minimized to below 0.6 s, which is much faster than most hydrogel-based actuators.

The pneumatic system is a typical actuation mode suitable for producing reversible snap-through deformation. Unlike the aforementioned strategies that rely on the stimuli-induced size change of embedded active materials to overcome the energy barrier, the pneumatic-powered control system can switch between different configurations by injecting air into associated channels. Tang et al. created a spine-inspired pneumatic soft robot by harnessing tunable snap-through bistability, which greatly improved the mechanical performance [62]. As shown in Figure 9c, this bistable hybrid soft actuator was composed of spring-based bistable linkages as the “skeletal spine” and pneumatic bending actuators as the “skeletal muscle.” The amplified performances of the actuator were attributed to the pre-stretching of the embedded spring, providing rapid energy storage and release as a force amplifier. Based on the reversible bending of the bistable actuator, they designed a high-speed crawling robot with a 20 times faster response speed (response time: tens of milliseconds) and over 3 times higher output force, compared to conventional soft crawlers. Faber et al. printed a muscle-free pneumatic-driven gripper for soft robotic actuation [63]: the outer surface of the gripper was equipped with an array of geometric concave domes, which could be individually pneumatically inverted. Upon 1.2 bar pressurization of the chamber, the gripper quickly jumped to the tightened state, with the gripping distance reduced by 79%. This gripper can sustain the actuating state even after the pressure is removed. Such a working principle for grabbing processes (Figure 9d) highlights the structure-based actuator design, which is applicable for different materials at multiple scales. Inspired by the prey-trapping strategy of the Venus flytrap, Lin et al. designed a high-speed soft gripper using snap-through instability to achieve a fast response to external stimuli. The pneumatic control method was adopted to make the trigger process repeatable, which can also actively control the trigger sensitivity [64].

### 3.4. Structural Bistability in Mechanical Metamaterials

**Figure 10:**
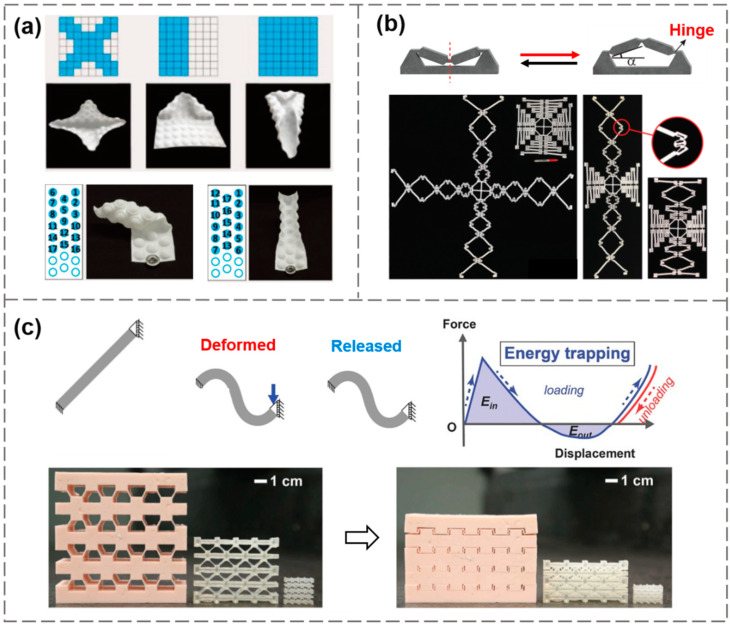
Using bistable building blocks to design mechanical metamaterials. (**a**) Dome-patterned shape-morphing metamaterials with digital programming. Reproduced from Ref. [63]; (**b**) A deployable structure consisting of serially connected bistable hinges. Reproduced from Ref. [96]; (**c**) Energy-absorbing metamaterials with architected bistable units. Reproduced from Ref. [97].

Mechanical metamaterials are a class of artificial materials with unconventional mechanical properties, such as negative Poisson’s ratio [91], negative swelling ratio [92], energy absorption [93], reconfigurable deployment [94], and a tunable phononic response [95]. These extraordinary functionalities are not directly related to the intrinsic properties of their constituent materials, but are dependent upon the elaborately designed microstructures as the basic building blocks. Elastic instability and bistable/multi-stable structures have been widely used in the design of these metamaterials. For example, Faber et al. proposed a novel shape-morphing metamaterial using snap-through bistability [63]. As illustrated in Figure 10a, a planar sheet with a dome-patterned array and the combination of the bistable states at individual domes can be programmed, giving rise to multi-stable configurations. This method established a mapping relationship between the local state of the bistable units and the global geometry of the entire structure. By controlling the specific domes to change into their inverted state, a distinct structure-associated shape morphing can be generated and switched. Interestingly, even with the same dome pattern, the loading history can influence the final configuration of the structure, and such path-dependency enriches the diversity of the shape-reconfigurable metamaterial.

The feature of bistable or multi-stable structures that can rest in any of the local equilibrium states without external inputs provides a structure-based self-locking mechanism, which differs from the energy storage caused by ordered or disordered arrangements of materials through phase transitions. Haghpanah et al. realized deployable metamaterials with multiple bistable units [96], each unit consisting of a relatively thick base and two inclined hinged beams, as shown in Figure 10b. When a downward vertical force was applied, the two inclined beams were pressed against each other, and beyond a critical force, the triangular frame snapped through and collapsed into the second stable position. By assembling multiple bistable units in series, a highly expandable lattice structure can be obtained under a relatively small applied load, with a significant expansion of more than three times of the original size. Shan et al. employed a mechanism to store elastic energy through the deformation of a titled beam that can transit between two stable configurations, triggered by the imposed displacement at one of its ends [97]. The mechanical response of these bistable beams was reversible and repeatable, usually independent of scale, rate, and loading history. As shown in Figure 10c, examples of structures at different length scales were fabricated by the DIW printing method for the impact experiments. This structural energy-trapping mechanism can be combined with the dissipative mechanisms of materials, such as viscoelasticity, to further enhance the impact resistance and protection of the system by harnessing multiple dissipation mechanisms.

## 4. Conclusions

Most of the existing studies focused on the spatial arrangement design of smart materials to obtain abundant shape-morphing modes. Our review not only summarized this area (Section 2), but also highlighted the advantages of using the snap-through bistability principle in shape-morphing (Section 3). We described each of the two approaches in detail and provided representative examples on how to integrate them to better program the shape-morphing. The following conclusions can be drawn:

- Mismatch strains caused by in-plane or through-thickness heterogeneity of material composition can generate two fundamental deformation modes, buckling and bending, which can be combined to achieve complex deformations.
- Due to the intrinsic characteristics of soft materials, the mismatch-induced shape-morphing in soft materials typically exhibits slow locomotion speed and low mechanical strength, limiting their applications in many scenarios.
- As an alternative programming strategy of soft morphing structures/devices, structural bistability has been widely used in the design of soft actuators due to the fast snap-through transition between bistable states.
- During the snap-through transition, large-magnitude deformations or dramatic shape changes arise within a short period of time (tens of milliseconds) due to the sudden release of stored strain energy, resulting in relatively large output forces at relatively low energy inputs.
- Structural bistability also provides a self-locking mechanism to maintain the deformed shape without external input, which can be employed in programmable metamaterials.
- It would be promising to integrate strain mismatch and snap-through bistability strategies to increase the richness and utility of shape transformations for practical applications.

## 5. Future Prospects

Despite the tremendous progress involving strain mismatch and snap-through bistability to program soft shape-morphing systems, there are still some challenges and issues to be addressed in the future, as outlined below:

- Reasonable layout design of responsive materials in bistable structures is still difficult and lacks effective theoretical and numerical guidance. Spatial arrangements such as proportions and positions of active and passive components can greatly affect the trigger sensitivity of snap-through movements.
- The selected smart materials should generate sufficient energy output under external stimuli to overcome the energy barrier. The development of new soft materials with high energy and power densities is of particular importance.
- Existing studies mostly focused on simple bistable structures, such as beams and shells, with little effort made to explore multi-stable structures with multiple equilibrium configurations that offer more shape transformation possibilities, which may endow actuators with richer working modes.
- When integrating a certain number of binary bistable units to construct a multi-stable system, it is challenging to use a single (or fewer) actuation input without sacrificing the rich reconfigurability. As the number of units increases, the individual control and sequential actuation of each bistable unit become intricate.
- Making the snap-through actuation repeatable and reversible is the key to transforming shape changes into a continuous motion. The current solution is to adopt pneumatic control systems, but this is not suitable for smart material systems.

## Figures and Tables

**Table 1:** Summary of representative shape transformations based on different design strategies, along with their characteristics and applications.

Design Strategy	Material	Morphing Mode	Stimulus	Characteristics: Size and Response Time	Application	Ref.
Mismatch strain (heterogeneous material composition)	Hydrogel	Rolling (buckling)	Swelling	500 × 300 × 12 μm, 30 min	Self-folding micro-device	[57]
	SMP	Worm-like shape change	Heat	20 × 25 × 0.12 mm, 90 s	Biomimetic 4D transformation	[58]
	Hydrogel	Folding	Swelling	700 × 700 × 1.5 μm, 10 min	Microscale reconfigurable origami structure	[59]
	LCE	Bending	Electric field	35 × 3 × 0.22 mm, 5 s	Soft actuator and robotic programmability	[60]
	Hydrogel	Twisting	Swelling	60 × 10 × 1 mm, 50 s	Soft gripper	[61]
Snap-through bistability	DE, polyimide, polyester	Snapping of 2D membrane	Electric field	60 × 15 × 0.55 mm, 1.7 s	Soft bistable actuator	[33]
	Hydrogel, PDMS	Buckling of 1D beam	Swelling	7 × 0.7 × 0.25 mm, 0.6 s	Logic and autonomous actuation	[54]
	Polylactic acid, Ecoflex	Bending	Pneumatic control	80 × 60 × 15 mm, 0.2~1 s	Soft robot with fast locomotion and high manipulation strength	[62]
	Ecoflex	Complex and multistep deformation	Mechanical manipulation/pneumatic control	~10 × ~10 × (0.5~1.4) mm, <0.1 s	Soft gripper, programmable metamaterial, and logic gate	[63]
	Polyurethane system, polycarbonate	Bending	Pneumatic control	100 × 20 × 10 mm, 0.8 s	Autonomous clamping actuator	[64]

## Data Availability

Not applicable.
